# Allergen-dependent oxidant formation requires purinoceptor activation of ADAM 10 and prothrombin

**DOI:** 10.1016/j.jaci.2016.12.954

**Published:** 2017-06

**Authors:** Jie Chen, Jihui Zhang, Theresa Tachie-Menson, Neha Shukla, David R. Garrod, Clive Robinson

**Affiliations:** a Institute for Infection and Immunity, St George's, University of London, London, United Kingdom; b Faculty of Life Sciences, University of Manchester, Manchester, United Kingdom

To the Editor:

Group 1 allergens, exemplified by Der p 1, are the most significant triggers within the allergenic repertoire of house dust mite (HDM) proteins capable of eliciting the intracellular generation of reactive oxidant species (ROS) by airway epithelial cells.^1^ This is because Der p 1, a cysteine peptidase, behaves as a prothrombinase, thereby triggering canonical activation of protease-activated receptor (PAR) 1 and 4 by thrombin.^1^ These events are preventable by Allergen Delivery Inhibitors or antagonists of PAR1 and PAR4 G-protein–coupled receptors.^1^ Intracellular ROS formation by any allergen is noteworthy because asthma is associated with deficits in antioxidant defences^1^ and ROS promote inflammation through transcription factor regulation, histone modifications, and the direct activation of signal transduction. The partially delineated pathway that leads to ROS production by HDM allergens converges with signaling from the ligation of Toll-like receptor 3 or melanoma differentiation–associated protein-5, which are key in host responses to respiratory viruses associated with asthma exacerbations.^1^ This convergence opens pannexons, releasing ATP, which is essential for allergen and viral RNA-dependent ROS production.^1^ Other pertinent effects of ATP include stimulation of IL-33 release, T_H_2 bias in dendritic antigen presenting cells, mast cell activation, and dyspnea.

“Sheddase”-dependent activation of epidermal growth factor receptor is implicated in G-protein–coupled receptor crosstalk, so we explored whether HDM allergen-dependent ROS generation requires the participation of sheddase metalloenzymes, especially those of the a disintegrin and metalloprotease (ADAM) family.

To investigate the production of intracellular ROS, we loaded human airway epithelial cells with dihydrorhodamine 123 and exposed them to a natural mixture of *Dermatophagoides pteronyssinus* allergens or 2′(3′)-*O*-(4-benzoylbenzoyl)adenosine 5′-triphosphate (BzATP) and uridine 5′-triphosphate (UTP) (to mimic the activation of P_2_X_7_ and P_2_Y purinoceptors by endogenously-released ATP) (see the Methods section in this article's Online Repository at www.jacionline.org).

Exploration of metalloenzymes capable of ectodomain cleavage or regulated intracellular proteolysis was prompted by the finding that epidermal growth factor receptor signaling is crucial for ROS generation in cells stimulated by HDM allergens, BzATP or UTP (see Fig E1, *A*-*C*, in this article's Online Repository at www.jacionline.org). The metalloenzyme inhibitors marimastat and TAPI-1 (N-[(2R)-2-[2-(hydroxyamino)-2-oxoethyl]-4-methyl-1-oxopentyl]-3-(2-napthalenyl)-1-alanyl-N-(2-aminoethyl)-1-alaninamide acetate) blunted ROS production by either HDM allergens or BzATP (see Fig E2, *A*-*D*, in this article's Online Repository at www.jacionline.org). Surprisingly, TAPI-2 (N-(R)-(2-(hydroxyaminocarbonyl)methyl)-4-methylpentanoyl-L-t-butyl-glycine-L-alanine 2-aminoethyl amide acetate) (which has greater selectivity than TAPI-1 for the “classical” sheddase ADAM 17) did not affect responses to BzATP, although it was an effective inhibitor of mixed HDM allergens (see Fig E2, *E* and *F*). From these results, and consistent with additional data (see Fig E3, *A*-*D*, in this article's Online Repository at www.jacionline.org), we inferred that ROS production involved a metalloprotease component distinct from ADAM 17.

Unexpectedly, the potent and selective ADAM 10 inhibitor, GI 254023X, attenuated intracellular generation of ROS by HDM, and was particularly efficacious in cells stimulated by BzATP or UTP (Fig 1, *A*-*C*), whereas it lacked effect in quiescent cells. Substantial involvement of ADAM 10 in responses to all 3 stimuli was confirmed by siRNA knockdown (Fig 1, *D*-*F*). As further proof, exogenously added recombinant human ADAM 10 elicited concentration-dependent ROS generation, which was inhibited by GI 254023X, thus authenticating its action (Fig 2, *A*-*C*). The effect of ADAM 10 was sensitive to AG 1478, confirming a receptor tyrosine-kinase–dependent component of the activation cycle (Fig 2, *D*).

**Fig 1:**
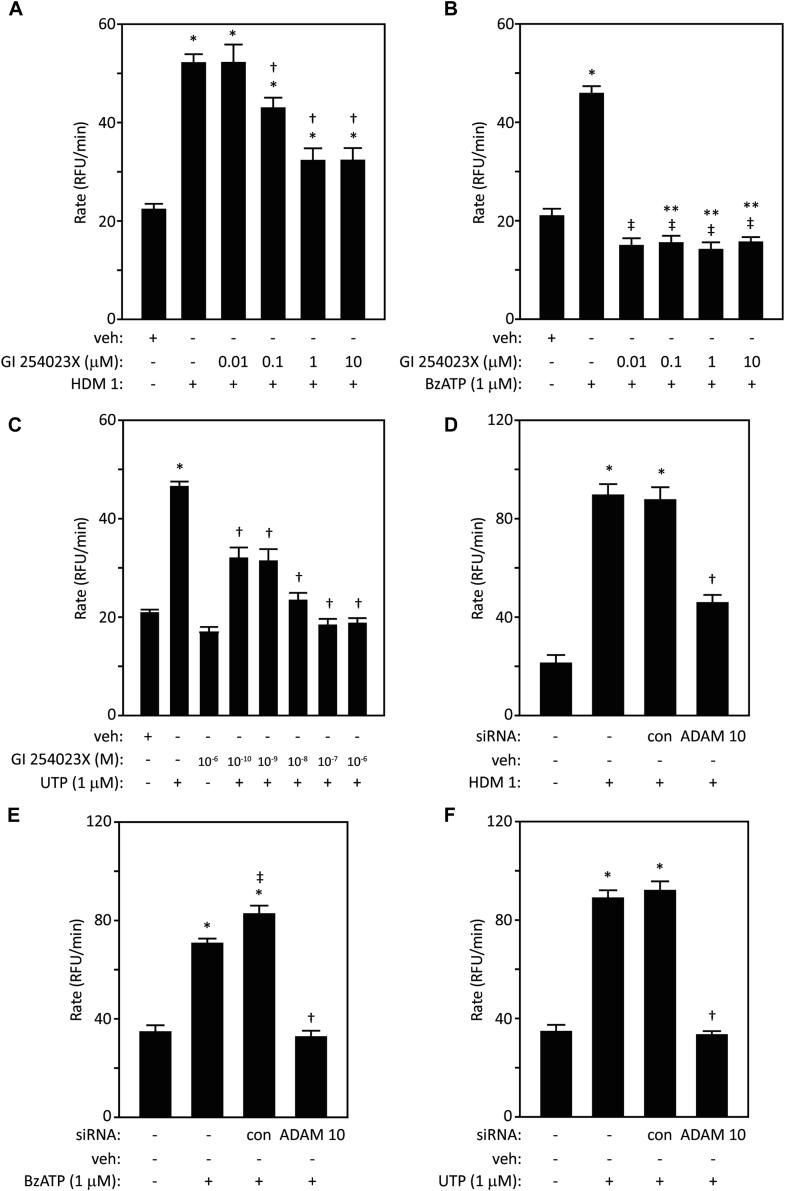
Inhibition by GI 254023X suggests that ADAM 10 is a mediator of intracellular ROS production by **(A)** HDM allergens (*P < .001 vs vehicle [veh], †*P* < .05-.001 vs HDM 1), **(B)** BzATP (*P < .001 vs veh, ‡*P* < .001 vs BzATP, **P < .05 vs veh), **(C)** UTP (*P < .001 vs veh, †*P* < .001 vs UTP). **D-F,** ADAM 10 gene silencing also reduces these responses (*P < .001 vs veh, †*P* < .001 vs HDM 1, BzATP, or UTP with or without control transfection, ‡*P* < .05 vs BzATP). *RFU*, Relative fluorescence units.

**Fig 2:**
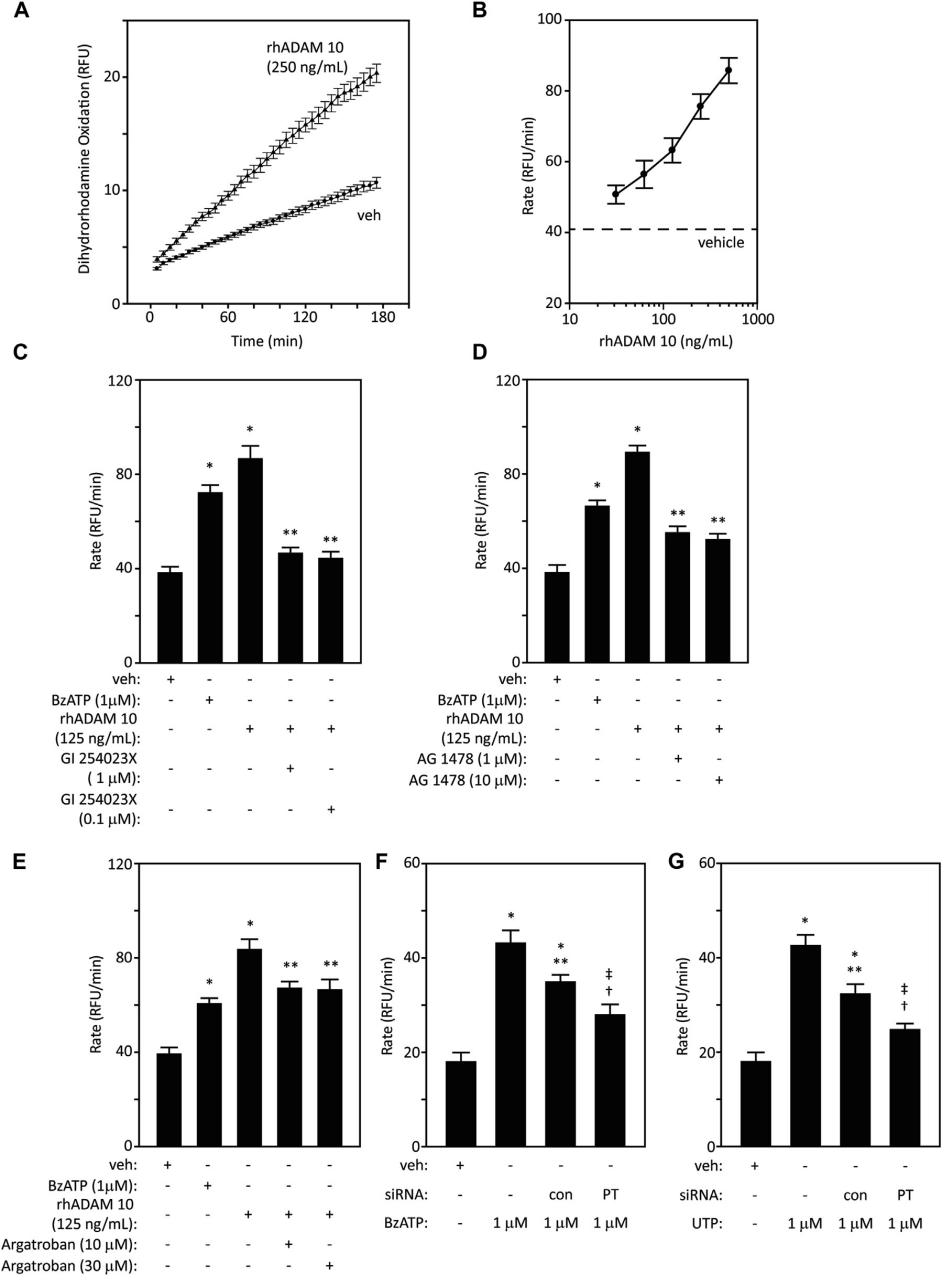
Recombinant human (rh) ADAM 10 stimulates intracellular ROS formation in airway epithelial cells. **A** and **B,** Progress curves and concentration-response relationship for dihydrorhodamine oxidation following vehicle (veh) or rhADAM 10. All concentrations *P* < .001 with respect to the dashed line. **C-E,** Inhibition by GI 254023X, AG 1478, or argatroban, respectively, of responses to ADAM 10. BzATP is shown for reference (*P < .001 vs veh, **P < .001 vs ADAM 10, †*P* < .01 vs veh, ‡*P* < .001 vs veh). **F,** Gene-silencing prothrombin (PT) blunts the response to BzATP (*P < .001 vs veh, **P < .05 vs BzATP, †*P* < .05 vs BzATP stimulation in control transfection, ‡*P* < .001 vs BzATP stimulation). **G,** As in Fig 2, *F*, but stimulation by UTP (*P < .001 vs veh, **P < .001 vs UTP, †*P* < .05 vs UTP stimulation in control transfection, ‡*P* < .001 vs UTP). *RFU*, Relative fluorescence units; *rhADAM 10*, recombinant human ADAM 10.

Surprisingly, argatroban inhibited responses to rhADAM 10, implying the formation of thrombin (Fig 2, *E*). We have previously shown that Der p 1 is a prothrombinase,^1^ confirmed here by demonstrating that siRNA knockdown of prothrombin attenuated the response to mixed HDM allergens (see Fig E4, *A*, in this article's Online Repository at www.jacionline.org). Moreover, we have now found that prothrombin knockdown blunted the responses to BzATP or UTP (Fig 2, *F* and *G*). This is consistent with ADAM 10 activation, which we show to be downstream from purinoceptor stimulation, operating a pathway to enhance thrombin formation. The principle of metalloprotease-initiated thrombin formation and ROS production was further exemplified using the snake venom protease, ecarin, whose ability to activate prothrombin by proteolytic cleavage is well established from its use as a clinical diagnostic in the ecarin clotting test. Like ADAM 10, ecarin is a member of the M12B protease subfamily and comprises metalloprotease, disintegrin, and cysteine-rich domains. Like ADAM 10, ecarin is a potent generator of intracellular ROS (Fig E4, *B*). Detailed biochemical studies investigating the activation of prothrombin by ADAM 10 are underway and will be reported separately.

Our data implicate purinoceptor-dependent activation of ADAM 10 as a downstream effector of ROS production in an innate response to HDM allergens. Significantly, ADAM 10 establishes a signaling cycle capable of sustaining prothrombin activation after its initiation by group 1 HDM allergens.^1^ In addition, as the principal sheddase of the adherens junction protein, E-cadherin,^2^ activation of ADAM 10 has the potential to augment any dysregulation of the epithelial barrier arising from targeted cleavage of tight junctions by group 1 HDM allergens.^3^

These findings expand the growing pleiotropic role of ADAM 10 in allergy. Illustratively, ADAM 10 drives T_H_2 bias^4^ and promotes IgE synthesis by being a CD23 sheddase,^5^ an effect incidentally ascribed to Der p 1 itself.^3^ In airway epithelial cells, ADAM 10 liberates CCL20 (which recruits dendritic cells and T_H_17 cells and promotes mucus hyperplasia), CCL2 (chemoattractant for dendritic cells), CCL5 (eosinophil chemokine), CXCL8 (neutrophil chemokine), and CXCL16 (T-cell chemoattractant).^6^, ^7^ It is also involved in stem cell factor-dependent mast cell migration. ADAM 10 expression is upregulated in a model of asthma and on B cells in patients with allergy and in T_H_2-prone mice.^8^, ^9^ The combination of high ADAM 10 expression on B cells within a T_H_2 cytokine environment causes mimicry of disease pathophysiology, namely, mucus cell hyperplasia, airway constriction, inflammation, and IgE production, whereas development of these is attenuated in mice deficient in ADAM 10.^9^

Intriguingly, ADAM 10 is also the cellular receptor for *Staphylococcus aureus* α-hemolysin toxin,^2^ suggesting that ADAM 10–dependent responses to allergens and infections, both viral and bacterial, may represent a signaling nexus in chronic severe disease exacerbations, which merits further examination in the clinic.

Additional information is available (see this article's Methods, Results, and References section in the Online Repository at www.jacionline.org).

## Figures and Tables

**Fig E1:**
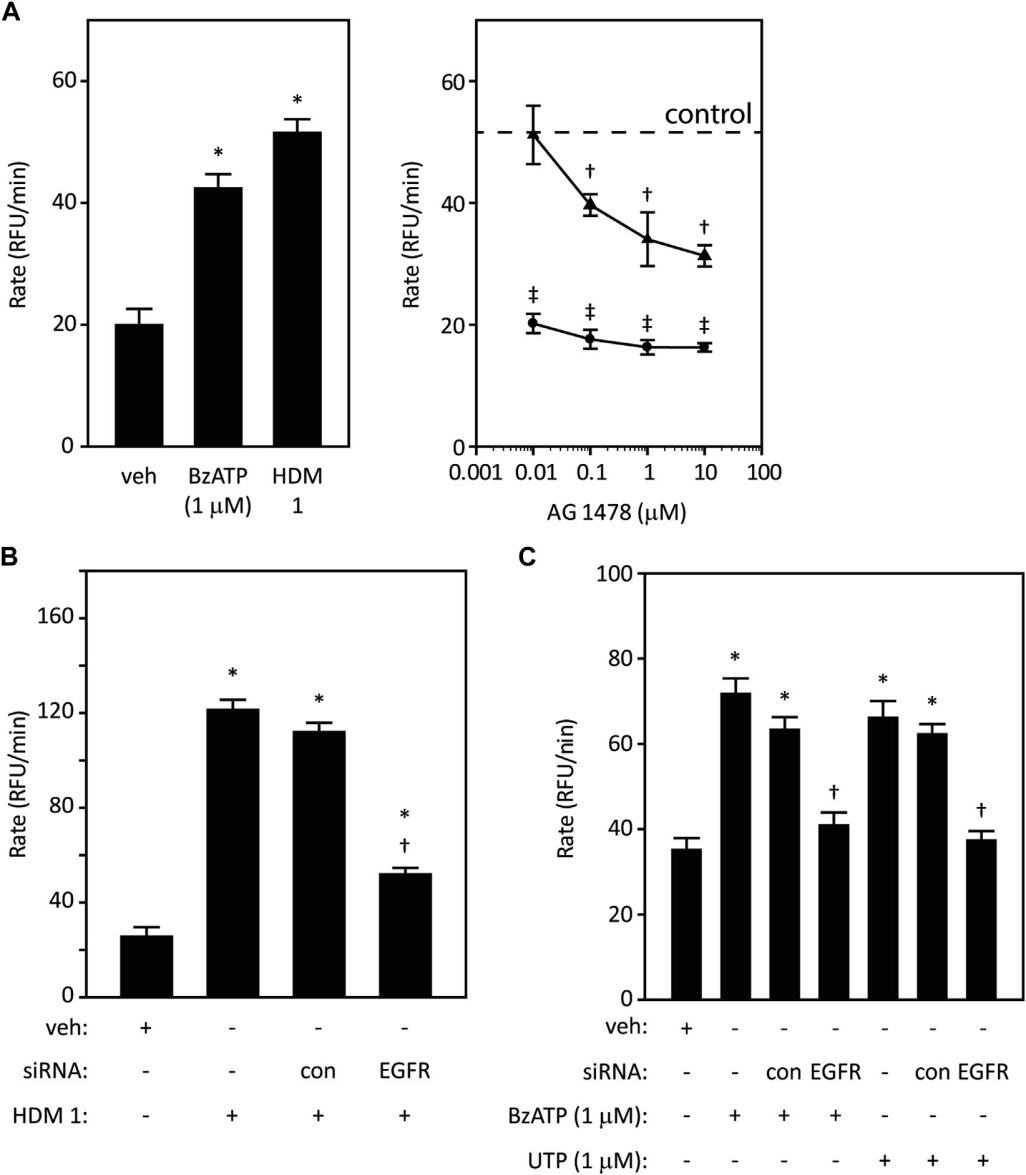
Generation of ROS by HDM allergens in airway epithelial cells is dependent on EGFR receptor tyrosine kinase signaling. **A,** Concentration-dependent inhibition by AG1478 of ROS production by HDM allergens *(triangles)* or BzATP *(circles)* (†*P* < .001 vs control HDM, ‡*P* < .001 vs control BzATP). **B,** EGFR-directed siRNA reduces the response to HDM allergens (*P < .001 vs vehicle [veh], †*P* < .001 vs HDM allergens in cells with or without control [con] transfection). **C,** Reduction in ROS generation activated by BzATP or UTP in cells treated with EGFR-directed siRNA (*P < .001 vs veh, †*P* < .001 vs BzATP or UTP with or without con transfection). *RFU*, Relative fluorescence units.

**Fig E2:**
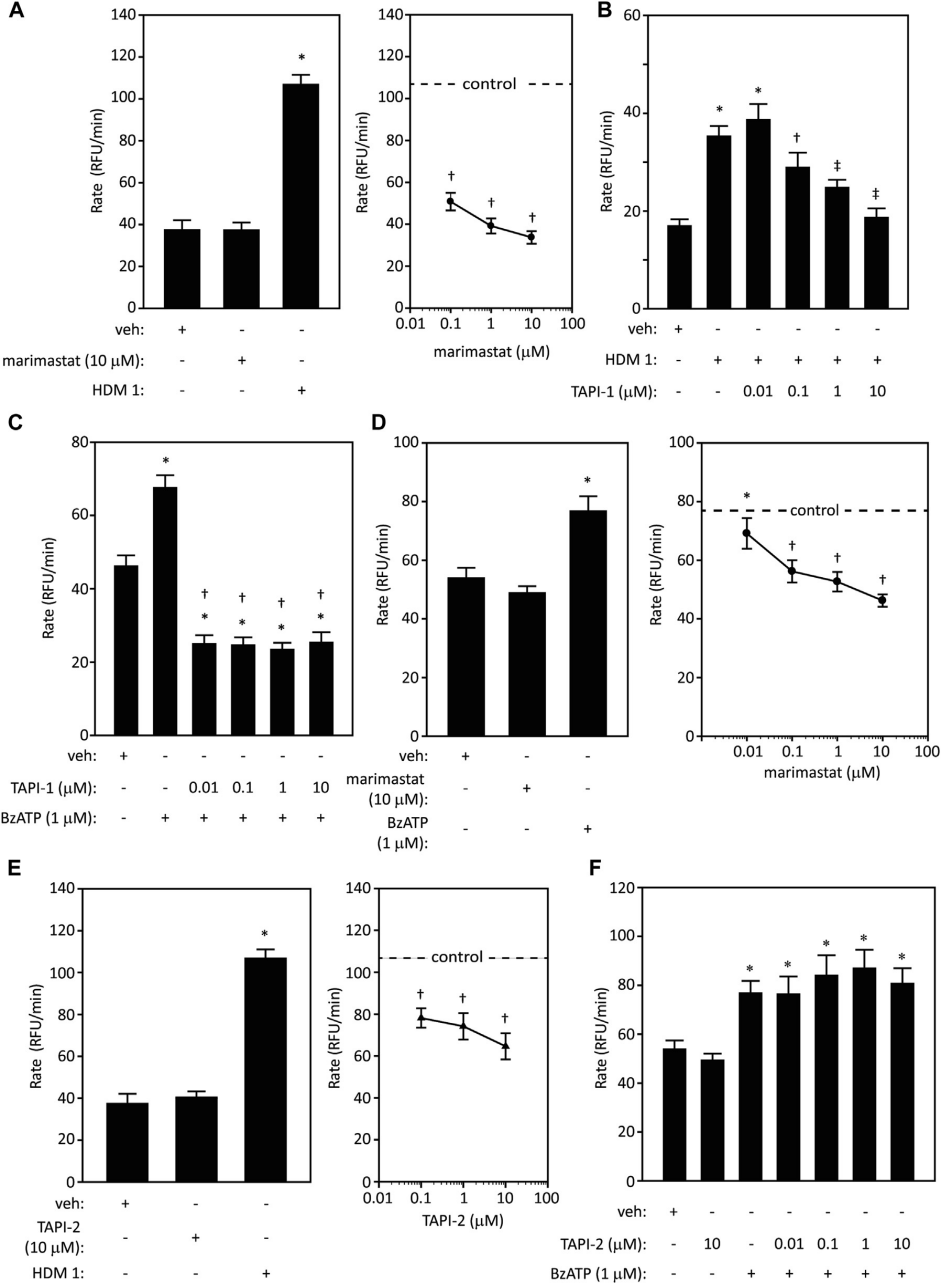
Effects of metalloprotease inhibitors on ROS generation in human airway epithelial cells stimulated by either HDM allergens or BzATP. **A,** Marimastat has no effect on baseline ROS production *(left panel)* but inhibits the response to HDM allergens *(right panel)* (*P < .001 vs vehicle [veh] or marimastat control, †*P* < .001 vs cells activated with HDM in the absence of marimastat). **B,** Concentration-dependent inhibition of HDM responses by TAPI-1 (*P < .001 vs veh, †*P* < .05, ‡*P* < .001 vs HDM 1). **C,** Inhibition of BzATP by TAPI-1 (*P < .001 vs veh, †*P* < .001 *vs* BzATP). **D,** Inhibition of BzATP by marimastat (*P < .001 vs veh or marimastat in unstimulated cells, †*P* < .001 vs BzATP control). **E,** Inhibition of the response to HDM allergens by TAPI-2. The control response to HDM allergens is displayed in the left section as a bar graph with mean ± SE shown, and as a dotted line in the concentration-inhibition curve in the right section of this panel (*P < .001 vs veh or TAPI-2 control, †*P* < .001 vs HDM 1). **F,** Lack of effect of TAPI-2 on the response to BzATP (*P < .001 vs veh or TAPI-2 control). *RFU*, Relative fluorescence units.

**Fig E3:**
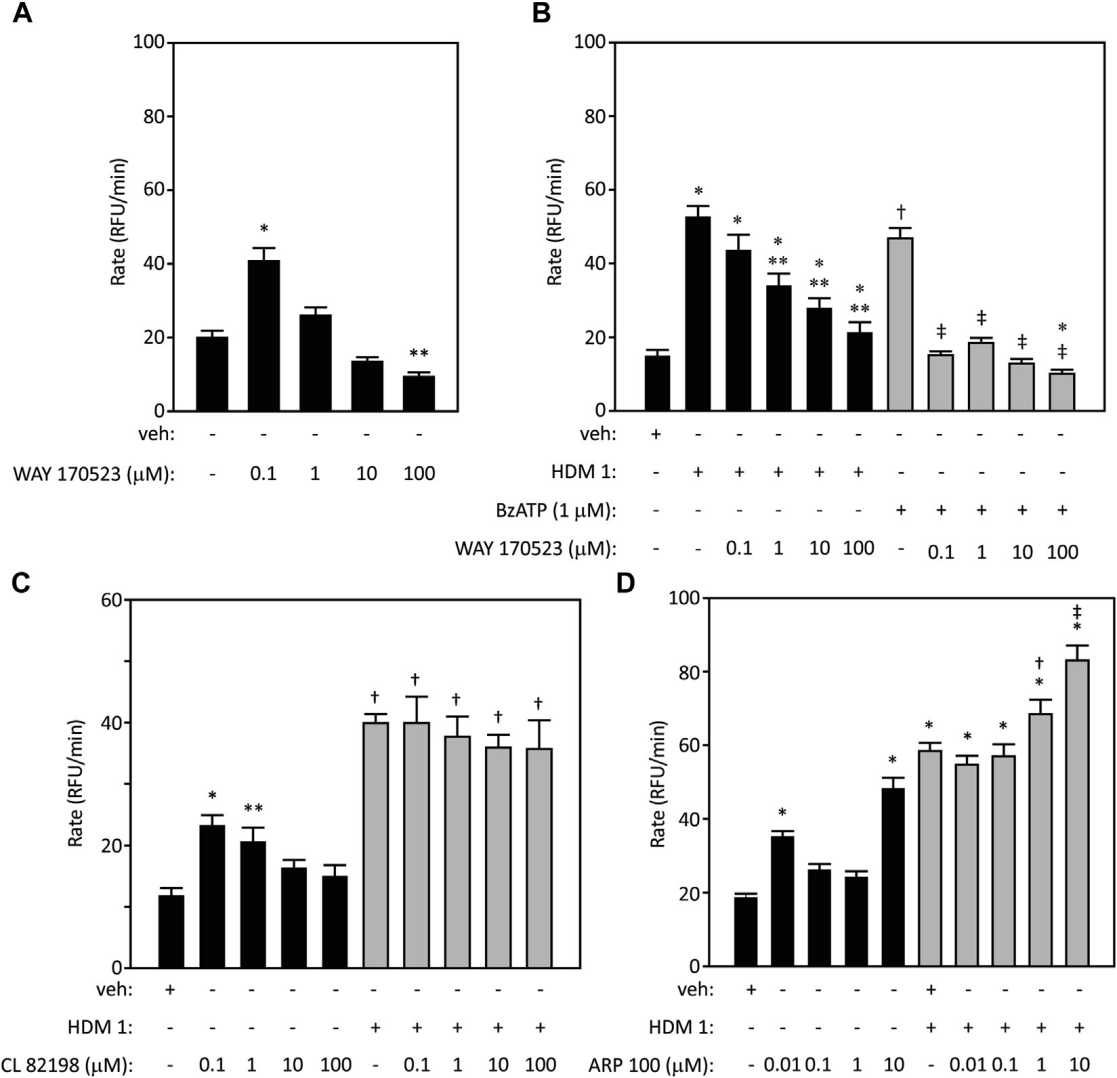
Effects of metalloprotease inhibitors on ROS generation activated by HDM allergens. **A,** Modulation of baseline ROS production in airway epithelial cells by WAY 170523 (*P < .001, **P < .01 vs vehicle [veh]). **B,** Inhibition of HDM allergen- or BzATP-dependent ROS production by WAY 170523 (*†*P* < .001 vs veh, **P < .05-.001 vs HDM, ‡*P* < .001 vs BzATP). **C,** Effect of CL 82198 on baseline ROS production and lack of action against HDM allergens (*P < .001 vs veh, **P < .05 vs veh, †*P* < .001 vs veh). **D,** Effect of ARP 100 on baseline ROS production and failure to inhibit the response to HDM allergens (*P < .001 vs veh, †*P* = .01 and ‡*P* < .001 vs HDM allergen). *RFU*, Relative fluorescence units.

**Fig E4:**
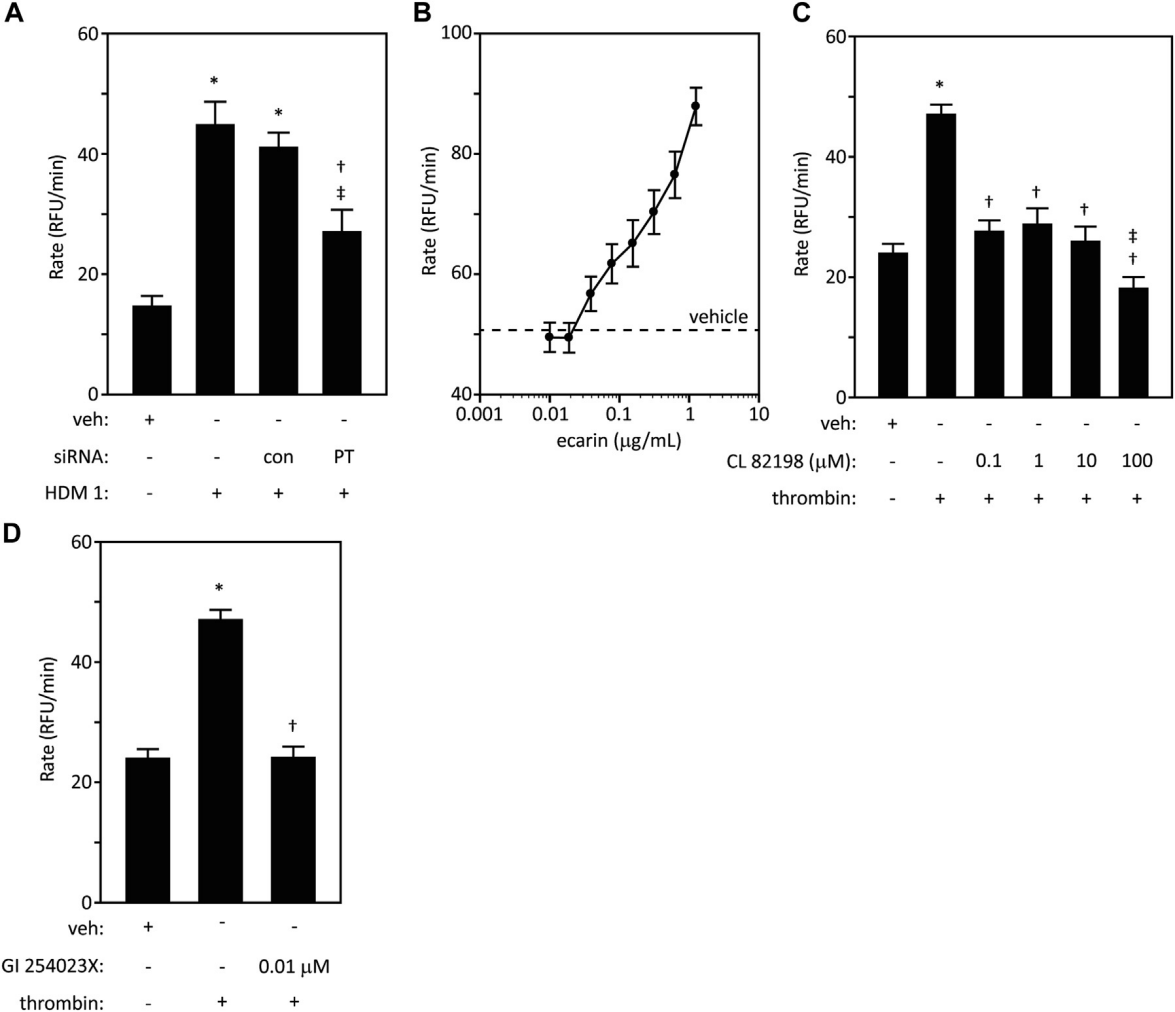
Thrombin dependency of intracellular ROS generation in airway epithelial cells. **A,** Knockdown of prothrombin (PT) expression by siRNA blunts the response to stimulation by mixed HDM allergens (*P < .001 vs vehicle [veh], †*P* < .05 vs HDM stimulation in cells with control [con] transfection, ‡*P* < .001 vs HDM stimulation). **B,** Concentration-dependent generation of ROS by the snake venom protease, ecarin. Dashed line shows ROS production rate in unstimulated cells. **C,** CL 82198 inhibits ROS generation by exogenous thrombin (*P < .001 vs veh, †*P* < .001 vs thrombin, ‡*P* < .05 vs veh). **D,** GI 254023X inhibits ROS generation by exogenous thrombin (*P < .001 vs veh, †*P* < .001 vs thrombin). *RFU*, Relative fluorescence units.

**Fig E5:**
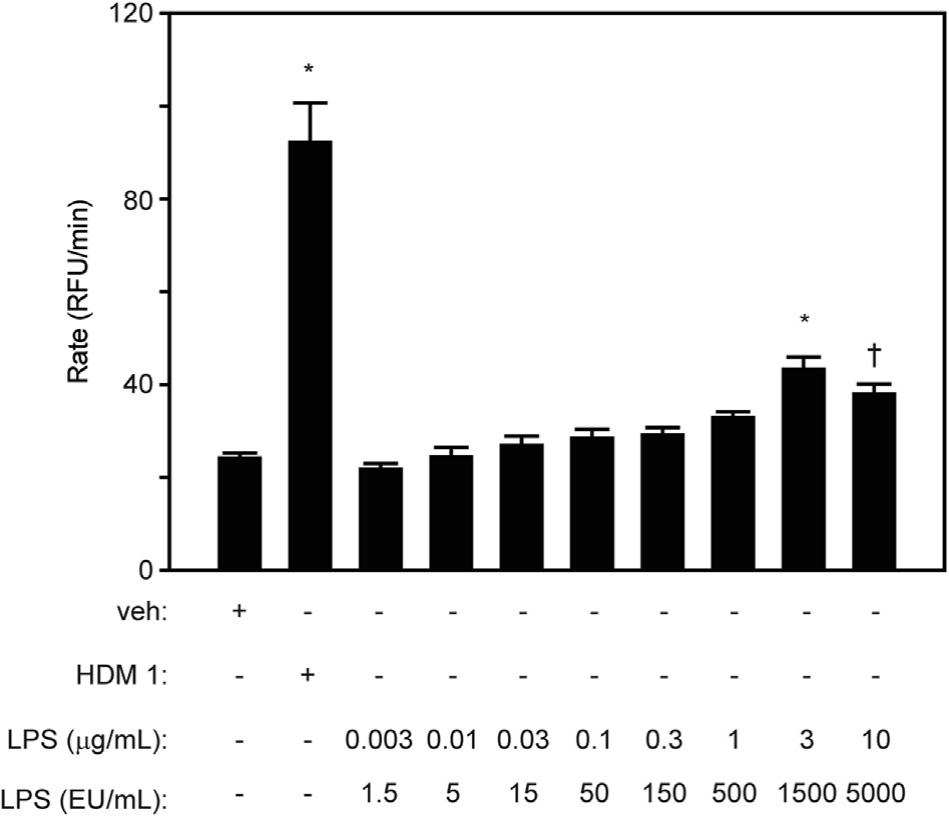
LPS fails to emulate intracellular ROS production by mixed HDM allergens in airway epithelial cells. (*P < .001 vs vehicle [veh], †*P* < .05 vs veh). LPS content of the mixed HDM preparation was 2.75 endotoxin unit/mL under the conditions of these experiments. *RFU*, Relative fluorescence units.

## Appendix

Group 1 allergens from HDMs form a novel subfamily of C1 cysteine peptidases differentiated by structural and functional differences in their zymogen prodomains.E1, E2 Persuasive evidence indicates that the proteolytic activity of these HDM allergens activates a mixture of physical events and innate immune responses that promote the development of persistent allergic sensitization to any allergen.E2, E3 This occurs because the proteolytic bioactivity nonselectively increases the probability of contact with dendritic antigen presenting cells and facilitates the polarization to allergic immunity through a combination of mechanisms: recapitulation of these processes maintains sensitization and promotes pathophysiological changes.^E2^

Group I allergens from all species of HDM have a highly conserved sequence identity such that they could be considered as a single drug target in the design of first-generation Allergen Delivery Inhibitor drugs directed against their cysteine protease activity.^E4^ Der p 1 from *D pteronyssinus*, commonly used as the general exemplar of these allergens, has recently been identified as the dominant component in the allergenic repertoire of HDM that stimulates the formation of superoxide anion (O_2_^−^) in mitochondria of human airway epithelial cells.^E5^ The production of this ROS is independent of IgE. Rather, it is part of a signaling cascade initiated by the conversion of prothrombin to thrombin by Der p 1, the consequent activation of PAR 1 and PAR 4 and the G-protein–coupled receptor signal transduction pathways coupled to them, the extracellular release of ATP, and the stimulation of purinergic signaling *inter alia* through P_2_X_7_ receptors.^E5^

In the airways, peptidase allergen to ROS signaling may be relevant to the pathogenesis of asthma because ROS exert a T_H_2 bias to immune responses. This risk may be exacerbated by deficits in enzymatic and/or nonenzymatic antioxidant defences.E10, E6, E7, E8, E9 Furthermore, components of the signaling mechanism activated by Der p 1 (eg, ATP and thrombin) are themselves implicated as primary transducers of innate immunity, which predispose to the development of acquired T_H_2 responses,E11, E12, E13, E5 and/or they underlie fundamental aspects of asthma pathophysiology.E14, E15, E16, E17 Illustratively, thrombin—which is present in asthmatic airways in elevated amountsE18, E19—is mitogenic in airways smooth muscle, is profibrogenic, and fosters the T_H_2 polarization of responses in dendritic antigen presenting cells.E14, E18 Also present in asthmatic airways at elevated concentration is ATP,^E12^ which has wide-ranging effects beyond the capacity to evoke neurogenic bronchoconstriction and dyspnea.^E20^ For example, ATP stimulates the release of IL-33 from airway epithelial cells,^E13^ augments IgE-dependent mediator release from mast cells,^E21^ and is another factor known to activate dendritic antigen presenting cells with a T_H_2 bias.^E12^ Moreover, intracellular ROS themselves have established roles in upregulating the expression of proinflammatory genes through operation of redox-sensitive transcription factors such as nuclear factor κappa B, AP-1, and Sp1,^E22^ histone modification and activation of signaling via members of the mitogen-activated protein kinase, and signal transducers and activators of transcription families.^E6^

In the experiments described here, we investigated the role of endogenous epithelial proteases in the signal transduction pathway leading to ROS production by Der p 1. Our specific focus was the role of zinc metalloproteases, especially ADAM enzymes. This supplementary information provides background data supporting the disclosure made in the accompanying “Letter to the Editor.”

## Methods

### Chemicals and reagents

ARP 100 (2-[((1,1′-biphenyl)-4-ylsulfonyl)-(1-methylethoxy)amino]-*N*-hydroxyacetamide), CL 82198 (*N*-[4-(4-morpholinyl)butyl]-2-benzofurancarboxamide hydrochloride), GI 254023X ((2R)-*N*-[(1S)-2,2-dimethyl-1-[(methylamino)carbonyl]propyl]-2-[(1S)-1-(*N*-hydroxyformamido)ethyl]-5-phenylpentanamide), WAY 170523 (*N*-[2-[4-[[[2-[(hydroxyamino)carbonyl]-4,6-dimethylphenyl](phenylmethyl)amino]sulfonyl]phenoxy]ethyl]-2-benzofurancarboxamide), and AG1478 (*N*-(3-chlorophenyl)-6,7-dimethoxy-4-quinazolinanine hydrochloride) were obtained from Tocris (Avonmouth, Bristol, United Kingdom [UK]).

TAPI-1 acetate (N-[(2R)-2-[2-(hydroxyamino)-2-oxoethyl]-4-methyl-1-oxopentyl]-3-(2-naphthalenyl)-l-alanyl-N-(2-aminoethyl)-l-alaninamide acetate salt), TAPI-2 acetate (N-(R)-(2-(hydroxyaminocarbonyl)methyl)-4-methylpentanoyl-l-t-butyl-glycine-l-alanine 2-aminoethyl amide),argatroban monohydrate ((2R,4R)-1-[(2S)-5-[(aminoiminomethyl)amino]-1-oxo-2-[[(1,2,3,4-tetrahydro-3-methyl-8-quinolinyl)sulfonyl]amino]pentyl]-4-methyl-2-piperidinecarboxylic acid), marimastat ((2S,3R)-N4-[(1S)-2,2-dimethyl-1-[(methylamino)carbonyl]propyl]-N1,2-dihydroxy-3-(2-methylpropyl)butanediamide), BzATP (2′(3′)-O-(4-benzoylbenzoyl)adenosine 5′-triphosphate triethylammonium salt), UTP (uridine 5′-triphosphate trisodium salt dehydrate), LPSs from *Escherichia coli* 0111:B4, and human thrombin were from Sigma-Aldrich (Poole, Dorset, UK). Dihydrorhodamine-123 was obtained from Life Technologies (Paisley, Renfrewshire, UK).

Cell culture media and reagents were obtained from Life Technologies, Sigma-Aldrich, and GE Healthcare (Little Chalfont, Buckinghamshire, UK). Transfection reagents and siRNA duplexes (typically mixtures of 3 target-specific 19-25 nt siRNAs or scrambled controls against no known targets) were obtained from Santa Cruz Biotechnology (Dallas, Tex). Baculovirus-derived, rhADAM 10 (Thr^214^ – Glu^672^) expressed in sf21 cells was obtained from R&D Systems (Abingdon, Oxfordshire, UK). Ecarin from *Echis carinatus* venom and thrombin from human plasma were supplied by Sigma-Aldrich.

Mixed, native HDM allergens in their natural proportions were prepared from laboratory cultures of *D pteronyssinus* according to our standard procedures. Der p 1 content of the allergen extracts was determined by ELISA (Indoor Biotechnologies, Cardiff, UK), whereas functional catalytic activity of Der p 1 was determined fluorimetrically using a Der p 1–selective substrate as described elsewhere.E4, E5 HDM mixtures were normalized by reference to Der p 1 content expressed as μg/mL. Thus, HDM 1 refers to mixed HDM whose Der p 1 concentration is 1 μg/mL. Normalization was necessary because the extracts contain multiple components and batch-to-batch variation in activity is seen. Der p 1 is the most important normalization denominator in these studies because it is the principal active component responsible for intracellular ROS generation.^E4^ For consistent batch-to-batch proteolytic activity of Der p 1 in allergen preparations, experiments were conducted in the presence of 5 mM l-cysteine. Endotoxin content in allergen preparations was determined by kinetic chromogenic limulus amebocyte assay (Endochrome-K, Charles River Laboratories International, Inc, Wilmington, Mass) according to manufacturer instructions.

### Cell culture and transfection

Calu-3 cells, which are both well validated and a relevant cellular model for these investigations, were cultured according to our standard procedures as described elsewhere.E23, E24, E25 Our previous work has demonstrated that ROS generation by HDM allergen treatment of calu-3 cells is mechanistically similar to responses seen in primary cultures of human airway epithelium.^E5^ Knockdown experiments with siRNA duplexes were performed according to the supplier protocol optimized for these experiments.

Intracellular ROS production was studied in cells plated into 96-well format on clear-bottomed black culture plates (Corning, Amsterdam, The Netherlands). Cells were washed and then loaded for 15 minutes at room temperature with dihydrorhodamine-123 (10 μM) in PBS, after which excess probe was removed by washing and the PBS replaced by HBSS containing 20 mM HEPES. Where appropriate, cells were then treated with inhibitors for 20 minutes at 37°C before the addition of HDM allergen or receptor agonists. Reactions were started by the addition of an appropriate stimulant (mixed HDM allergens, purified Der p 1, rhADAM 10, or LPS) and maintained at 30°C under constant humidity in an Envision plate reader (Perkin Elmer, Seer Green, Buckinghamshire, UK) for the duration of the experiment. Fluorescence measurements were made every 5 minutes (excitation 485 nm, emission 535 nm), and the maximum rate of oxidant production determined from the progress curves (increase in fluorescence upon oxidation of dihydrorhodamine-123 to rhodamine) recorded for each well over a 2.5-hour period.

### Statistical analyses

Analyses were performed using SigmaPlot v12. Data are shown as mean ± SEM (n = 8) in single experiments, which were replicated more than 3 times. Significance was determined using 1-way ANOVA with Newman-Keuls *post hoc* testing.

## Results

### Modulation of intracellular oxidant formation by inhibition of metalloproteases and thrombin formation

To investigate the IgE-independent production of ROS by allergen exposure, cells were treated with a natural mixture containing all HDM allergens. To elucidate the role of ATP in signaling, we used BzATP and UTP to achieve stimulation of P_2_X_7_ and P_2_Y receptors, respectively. Responses to HDM and BzATP were inhibited by AG1478, which indicated a role for epidermal growth factor receptor (EGFR) signaling (Fig E1, *A*). This was confirmed by siRNA knockdown in cells stimulated with mixed HDM allergens, BzATP or UTP (Fig E1, *B* and *C*). Given that their involvement in the generation of EGFR agonists or other sheddable ligands is established, this led us to investigate the role of matrix metalloproteases (MMPs) and ADAMs in the signal transduction pathway(s) operated by HDM allergen stimulation.

Both marimastat and TAPI-1 were concentration-dependent inhibitors of ROS generation in airway epithelial cells stimulated by mixed HDM allergens or BzATP (Fig E2, *A*-*D*). In contrast, while being an effective inhibitor of HDM-evoked ROS formation (Fig E2, *E*), TAPI-2 was inactive when BzATP was the stimulant (Fig E2, *F*). These results suggested an involvement of multiple metalloproteases in ROS generation in airway epithelial cells, so studies were conducted to explore possible candidates.

Conventionally regarded as an inhibitor of MMP-13, MMP-9, ADAM 17, and, less effectively, MMP-1, compound WAY 170523 inhibited intracellular ROS production by both mixed HDM allergens and BzATP, whereas the selective MMP-13 inhibitor CL 82198 was ineffective (Fig E3, *A*-*C*). The effects of WAY 170523 showed complexity, however, in affecting baseline ROS production. At low concentrations of WAY 170523 this was stimulated, whereas the highest concentration suppressed ROS production below baseline (Fig E3, *B*). Human airway epithelial cells are sources of other metalloproteases, notably MMP-2, which is a target of marimastat, a broad-spectrum hydroxamate inhibitor. However, ARP 100, which has greater selectivity, did not attenuate responses in cells activated by mixed HDM allergens, thus excluding a significant role for MMP-2 in this pathway of intracellular ROS formation (Fig E3, *D*).

Confirming the essential role of thrombin formation in this pathway of ROS formation suggested by our previous work,^E5^ responses to HDM allergens were strongly attenuated in cells treated with siRNA duplexes targeting prothrombin (Fig E4, *A*). Additional studies revealed that treatment of cells with ecarin, a snake venom metalloprotease related to ADAM 10 and a known activator of prothrombin, was also a stimulus for ROS production, consistent with the principle that ADAM 10 (or its targets) may be a hitherto unrecognized endogenous activator of prothrombin (Fig E4, *B*). Although CL 82198 lacked effect against activation by mixed HDM allergens (Fig E3, *C*), it was an inhibitor of ROS production by exogenous thrombin (Fig E4, *C*). GI 254023X was a potent inhibitor of responses to exogenous thrombin (Fig E4, *D*), as anticipated from an involvement of ADAM 10 in the signaling pathway connecting thrombin generation by HDM to ROS production.

In contrast to stimulation by mixed HDM allergens, bacterial LPS was a weak activator of intracellular ROS production, with significant stimulation observed only at concentrations of more than 1000 endotoxin unit/mL (Fig E5).

## Discussion

In the associated Letter to the Editor and this accompanying supporting information, we provide the first evidence implicating ADAM 10 as a crucial component of innate immune responses that trigger the intracellular formation of ROS when airway epithelial cells are stimulated by HDM allergens from *D pteronyssinus*. As demonstrated in our previous work, this effect is due to the proteolytic activity of Der p 1 because it can be replicated by the purified allergen and blocked by potent, selective inhibitors specifically designed to target the proteolytic bioactivity of this allergen.^E5^ Bacterial LPSs, inevitably present in allergen mixtures derived from natural cultures and occurring as contaminants in purified allergens derived therefrom, are potent activators of ROS formation in numerous cell types. As further demonstration that HDM-induced generation of intracellular ROS in human airway epithelial cells is primarily dependent on the proteolytic bioactivity of Der p 1, we now show evidence that unambiguously demonstrates that LPS, when present at levels found in our allergen preparations, fails to evoke significant intracellular ROS activation.

The involvement of ADAM 10 in the pathway of HDM allergen-dependent intracellular ROS generation adds a further dimension to the emerging appreciation of this enzyme's role in the initiation and maintenance of allergy. In addition to these, another well-established target of ADAM 10, and also ADAM 17, is HB-EGF—notable for its roles in re-epithelialization and the regulation of mitogenesis in smooth muscle and fibroblasts.^E26^

Generation of intracellular ROS in airway epithelial cells by mixed HDM allergens requires the cysteine peptidase activity of group 1 allergens. These allergens are the binding targets of ADZ 51,457 and ADZ 51,529, compounds that are effective inhibitors of ROS formation and that exhibit anti-inflammatory activity *in vivo*.E4, E5 Previous work identified the initiating step in ROS production as the activating cleavage of prothrombin to thrombin by Der p 1.^E5^ Der p 1, and by reasonable inference all other group 1 mite allergens, can thus be regarded in this context as a prothrombinase. Thrombin then stimulates PAR1 and PAR4 to launch a cascade of signaling events, including ATP release, which culminate in the formation of ROS in mitochondria and nuclei.^E5^

Given that the generation of intracellular ROS by BzATP and UTP was also dependent on ADAM 10, it is reasonable to conclude that a significant role in ROS generation by this sheddase occurs downstream of pannexon channel opening and ATP release. This inference is supported by pharmacologic data with metalloprotease inhibitors. Although TAPI-2, a potent inhibitor with good selectivity for ADAM 17, attenuated ROS production by HDM allergen treatment, it was ineffective against BzATP. Moreover, metalloenzyme inhibitors (marimastat, TAPI-1, WAY 170523), which have less selectivity for ADAM 17, were surprisingly effective inhibitors of responses to both BzATP and HDM allergens.

The unexpected contribution of ADAM 10 to intracellular ROS formation was revealed by demonstrating that pharmacological inhibition or knockdown of ADAM 10 reduced the responses to HDM allergens, BzATP and UTP. Exogenously added ADAM 10 elicited sustained ROS generation, which was inhibited by blockade of EGFR tyrosine kinase signaling. The ability of EGFR signaling inhibition or EGFR knockdown to strongly attenuate ROS generation by HDM allergens, BzATP and UTP, together with evidence that both ADAM 10 and ADAM 17 contribute to ROS generation suggests that the mechanism involved is the hub of an innate signaling network comprising several pleiotropic elements.

Our data suggest that ADAM 17 activation and at least 1 instance of EGFR signaling probably lie upstream from the opening of pannexon channels, whereas ADAM 10 and further EGFR signaling lie downstream. Receptor crosstalk between EGFR and G-protein—coupled receptors is well documented and, specifically, a precedent for PAR-EGFR cross-talk exists.^E27^ Thus, given our earlier findings,^E5^ ADAM 17/EGFR-dependent events may be closely coupled to the thrombin-mediated activation of PAR 1 and PAR 4 by Der p 1, through intramembrane events and/or an increase in intracellular calcium arising from PAR activation. In the case of ADAM 10, whose ligand shedding activity is also calcium dependent,E28, E29 the calcium signal may partly depend on the opening of the P_2_X_7_ channel and protein kinase C activation after the binding of ATP by P_2_X_7_ and P_2_Y receptors, respectively. Extracellular ATP is present at elevated levels in the airways in asthma and is a bronchoconstrictor that produces profound dyspnea.^E20^ It exacerbates IgE-mediated release of histamine, stimulates mediator release by eosinophils, and polarizes dendritic cells into T_H_2-directed responses.E11, E12, E21 The ability of Der p 1 to cause ATP release through an innate mechanism that exists alongside IgE-mediated responses suggests that investigation of the chronic corollaries of these innate effects may reveal new insights into the initiation of disease pathophysiology. The present studies delineate novel mechanisms whose further understanding will require appropriate preclinical and clinical *in vivo* models.

An anomaly noted during the pharmacological studies concerned the selective MMP-13 inhibitor, CL 82198. This was without effect on ROS production elicited by HDM allergen treatment, yet it was a potent inhibitor when responses were evoked directly by exogenous thrombin. Thrombin promotes MMP-13 expression,^E30^ which itself is a noncanonical activator of PAR 1 and an epithelial sheddase,E31, E32 so the differential sensitivity may therefore simply reflect mass action disparities. However, a reasonable inference from these experiments is that MMP-13 is another putative regulator of ROS production.

In summary, this study provides the first evidence for ADAM 10 proteolytic activity as an essential molecular component in the critical path that couples the innate detection of Der p 1 with the generation of intracellular and extracellular signals that have the capacity to initiate, develop, and maintain a predisposition to the immunological profile and pathophysiology of asthma. These findings invite the further clinical examination of these events, especially in the context of poor antioxidant status.
